# Leukotriene enhances NMDA-induced inward currents in dorsal horn neurons of the rat spinal cord after peripheral nerve injury

**DOI:** 10.1186/s12990-015-0059-5

**Published:** 2015-09-09

**Authors:** Yasukuni Kiyoyuki, Wataru Taniguchi, Masamichi Okubo, Hiroki Yamanaka, Kimiko Kobayashi, Naoko Nishio, Terumasa Nakatsuka, Koichi Noguchi

**Affiliations:** Department of Anatomy and Neuroscience, Hyogo College of Medicine, 1-1 Mukogawa-cho, Nishinomiya, Hyogo, 663-8501 Japan; Pain Research Center, Kansai University of Health Sciences, 2-11-1 Wakaba Kumatori-cho, Osaka, 590-0482 Japan; Department of Orthopedic Surgery, Wakayama Medical University, 811-1 Mimiidera, Wakayama, 641-8510 Japan

## Abstract

**Background:**

LTB4 is classified as a leukotriene (LT), a group of lipid mediators that are derived from arachidonic acid. It is recognized that leukotrienes are involved in the pathogenesis of many diseases, including peripheral inflammatory pain. However, little is known about the effects of leukotrienes on the spinal dorsal horn during neuropathic pain. Previously, we reported that there was increased expression of 5-lipoxygenase (5-LO) at spinal microglia, and the leukotriene B4 receptor 1 (BLT1), a high affinity receptor of LTB4, in spinal neurons in spared nerve injury (SNI) model rats. In the present study, we examined the effects of LTB4 on spinal dorsal horn neurons in both naïve and SNI model rats using patch-clamp methods.

**Results:**

Bath application of LTB4 did not change AMPA receptor-mediated spontaneous excitatory postsynaptic currents (sEPSCs) or membrane potentials. However, we found that LTB4 enhanced the amplitude of NMDA receptor-mediated sEPSCs and significantly increased exogenous NMDA-induced inward currents in SNI model rats. This increase of inward currents could be inhibited by a selective LTB4 antagonist, U75302, as well as a GDP-β-S, a G-protein inhibitor. These results indicate that both increased LTB4 from spinal microglia or increased BLT1 in spinal neurons after peripheral nerve injury can enhance the activity of NMDA receptors through intracellular G-proteins in spinal dorsal horn neurons.

**Conclusion:**

Our findings showed that LTB4, which may originate from microglia, can activate BLT1 receptors which are expressed on the membrane of spinal dorsal horn neurons during neuropathic pain. This glia-neuron interaction induces the enhancement of NMDA currents through intracellular G-proteins. The enhancement of NMDA receptor sensitivity of dorsal horn neurons may lead to central sensitization, leading to mechanical pain hypersensitivity.

## Background

Nociceptive pathways are recognized to be dynamically modulated by gene expression, protein synthesis, and intracellular signaling after peripheral nerve injury [1–3]. Particularly, activated glial cells in the spinal cord after peripheral nerve injury produce and release proinflammatory cytokines, such as interleukin-1 beta (IL-1β), tumor necrosis factor-alpha (TNF-α), and neurotrophins, resulting in the enhancement of excitability in nociceptive dorsal horn neurons [4–8]. Recently, lipid mediators, their receptors, and proinflammatory cytokines have become considered some of most interesting molecules in pain research [9–11]. Accumulating evidence suggests that lipid mediators, such as prostaglandins (PG), lysophosphatidic acid, platelet-activating factor, and their receptors, have critical roles in nociceptive pathways and pathological pain [12–14].

Leukotrienes (LTs) are a group of lipid mediators derived from arachidonic acid (AA). LTs include several products catalyzed in the 5-lipoxygenase (5-LO) pathway that are then released from the cell membrane. LTs have a variety of biological actions and have been recognized as important factors in numerous disease processes, including allergic diseases (e.g. asthma, atopic dermatitis), local or systemic inflammatory diseases (e.g. rheumatoid arthritis, psoriasis), cancer, and cardiovascular diseases [15, 16]. AA is converted to leukotriene A4 (LTA4) which is then converted to LTB4, LTC4, LTD4, or LTE4. These products are known as bioactive leukotrienes. LTC4, LTD4 and LTE4 are collectively termed ‘cysteinyl leukotrienes’ (CysLTs). LTs act by binding to specific receptors which are located on the outer plasma membrane of structural and inflammatory cells [15]. So far, four G-protein coupled receptors have been cloned and characterized as LTs receptors [17–20]. It is recognized that the LTB4 receptor 1 (BLT1) has a high affinity for LTB4, but that BLT2 has a low affinity for LTB4 and many other LTs. Studies have shown that lipid mediators have a key role in the pain mechanisms of peripheral inflammation, while other research indicates the involvement of spinal lipoxygenase metabolites in hyperalgesic responses [16]. For example, prostaglandin E2(PGE2) directly depolarizes spinal dorsal horn neurons via the prostaglandin E receptor 2 (EP2)-like receptor, resulting in the enhancement of dorsal horn neuronal excitability [9]. Lysophosphatidic acid may be released in the spinal cord after nerve injury and affect the excitability of dorsal horn neurons, which may be involved in hyperalgesia after peripheral nerve injury [12, 21]. Some studies indicate that LTs and their synthesizing enzymes are present in the central nervous system, including the spinal cord, and play important roles in both normal and pathological states [22–24].

Previously, we reported that BLT1 mRNA was expressed by non-neuronal cells in DRG and that CysLT2 mRNA was preferentially expressed by small DRG neurons [25, 26]. Furthermore, we showed that the expression of 5-LO in spinal microglia and BLT1 mRNAs in spinal neurons (especially laminae III–IV) was increased during peripheral nerve injury using immunohistochemistry, reverse transcription-polymerase chain reaction (RT-PCR), in situ hybridization histochemistry (ISHH), and behavioral experiments [25]. These findings suggest that LTB4 contributes to the central sensitization of the spinal cord after peripheral nerve injury. However, it is unclear how LTB4 acts on excitatory neurotransmission in the spinal dorsal horn. In the present study, we examine how LTB4 modulates excitatory neurotransmission in the spinal dorsal horn after nerve injury.

## Results

Whole-cell patch-clamp recordings were made from a total of 181 spinal dorsal horn neurons. Stable recordings were obtained from slices more than 12 h after the dissection, and recordings were made from single dorsal horn neurons for up to 4 h.

### BLT1, the receptor of LTB4, increased in laminae III-IV in SNI model rats

**Fig. 1:**
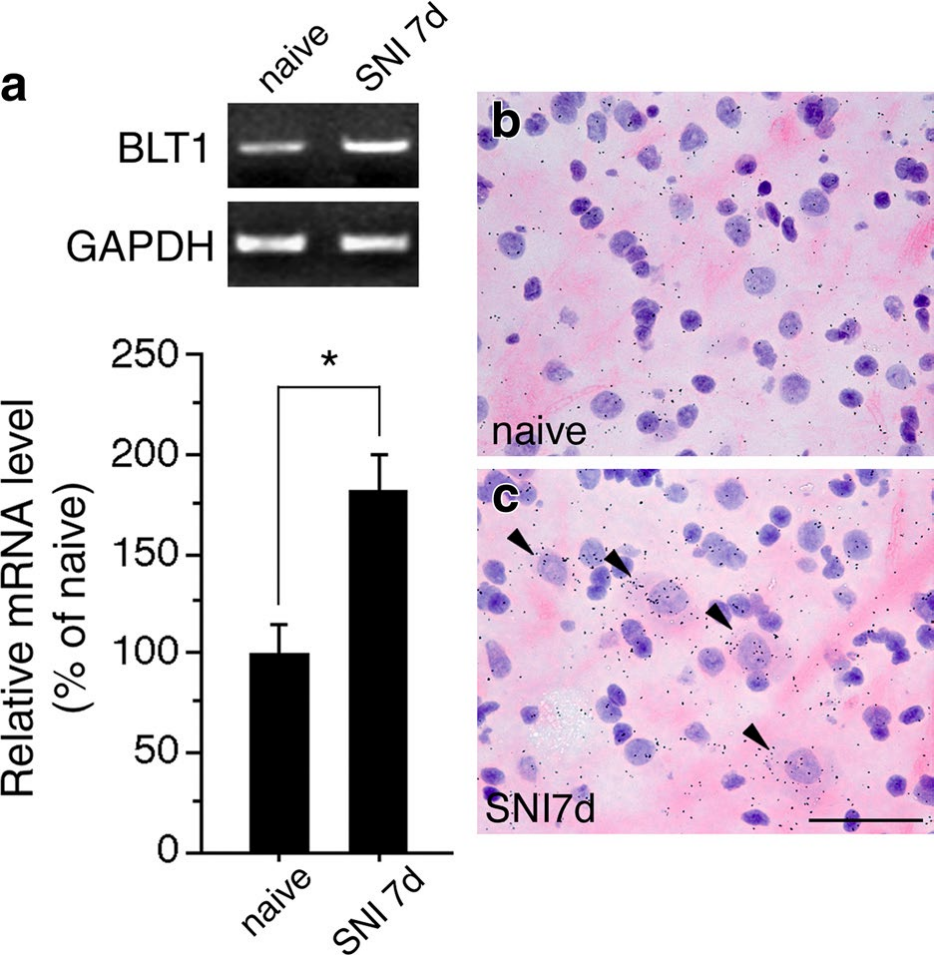
BLT1 mRNA increased in dorsal horn neurons after peripheral nerve injury. **a** Expression of BLT1 mRNA in the ipsilateral spinal cord following SNI surgery by conventional RT-PCR. *Upper gel panels* show PCR products from the dorsal horn of the L4–5 spinal cord taken from naïve and 7 days after SNI. Lower histogram shows the quantification of relative BLT1 mRNA levels (mean ± SEM; *, *P* < *0.05* compared with naïve). **b**, **c** Bright-field images of in situ hybridization showing BLT1 mRNA expression in lamina III of the spinal dorsal horn in naïve (**b**) and 7 days after SNI (**c**). *Solid arrowheads* indicate the positive cells. *Scale bar* 25 µm

In order to show that BLT1 mRNA expression is increased in the laminae after peripheral nerve injury, we performed RT-PCR and in situ hybridization histochemistry of dorsal horn tissue. We confirmed that SNI induced the expression of BLT1 mRNA significantly compared to naïve rats (Fig. 1a). We found a majority of neurons showing apparent increase of BLT1 mRNA were located in laminae III-IV (Fig. 1b, c). Thus, our next step was to examine the effect of LTB4 on laminae III–IV neurons using patch-clamp recording.

### LTB4 had no effect on AMPA receptor-mediated neurotransmission in rat spinal dorsal horn neurons

**Fig. 2:**
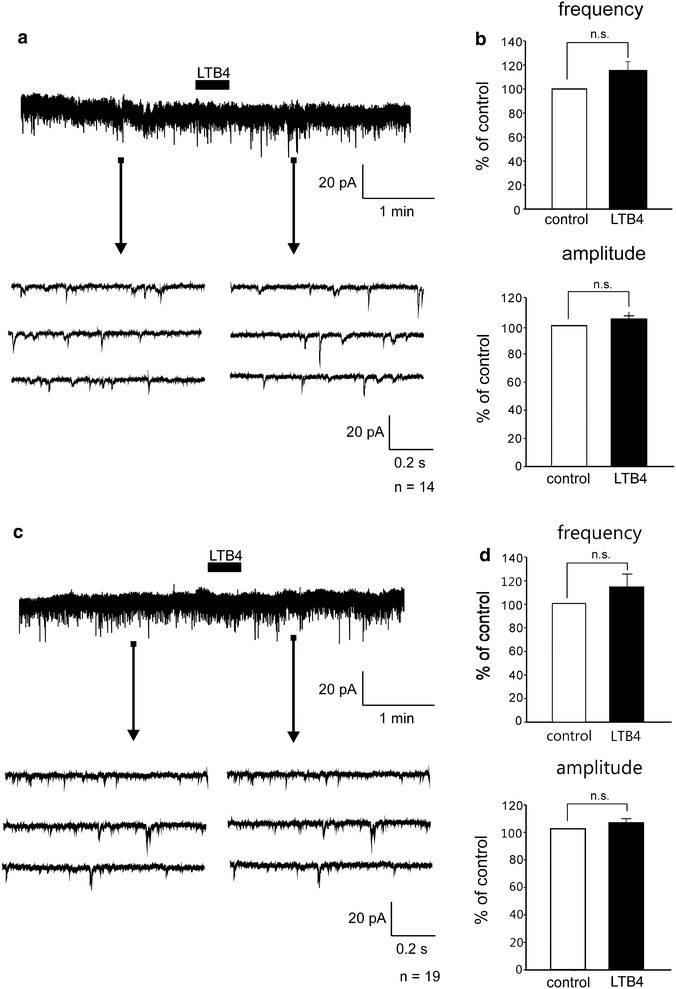
LTB4 had no effects on AMPA receptor-mediated sEPSC in spinal dorsal horn neurons. The frequency and amplitude of sEPSC were not affected by application of LTB4 in dorsal horn neurons of either naïve (**a**, **b**) or SNI model rats (**c**, **d**). *Bar* *graphs* showing the average frequency and amplitude of sEPSC under the treatment with LTB4 (*black bar*), relative to those before this treatment (control, *open bar*) in naïve (**b**) and SNI model rats (**d**) (naïve; n = 14. SNI; n = 19, mean ± SEM). n.s., not significant

All the recorded neurons that were tested exhibited spontaneous excitatory postsynaptic currents (sEPSCs) at a holding potential (VH) of −70 mV, at which no inhibitory postsynaptic currents (IPSCs) were observed because the reversal potential for IPSCs was near −70 mV [27]. To examine the effects of LTB4 on α-amino-3-hydroxy-5-methyl-4-isoxazolepropionic acid (AMPA) receptor-mediated excitatory synaptic transmission, LTB4 (10 μM) was dissolved in Krebs solution and was applied by perfusion for 1 min. When the cell membrane is fixed at −70 mV, *N*-methyl-d-aspartate (NMDA) receptors are blocked by Mg^2+^. The frequency and amplitude of sEPSC were not affected by application of LTB4 in dorsal horn neurons (n = 14) of naïve rats (Fig. 2a). The frequency and the amplitude were, respectively, 116.1 ± 7.4 % (n = 14, *P* > 0.05) of control (1.2 ± 0.4 Hz) and 105.2 ± 3.9 % (n = 14, *P* > 0.05) of control (8.5 ± 1.8 pA, Fig. 2b). The frequency and amplitude of sEPSC also were not affected by the application of LTB4 in dorsal horn neurons (n = 19) of spared nerve injury (SNI) model rats (Fig. 2c, d). The frequency and the amplitude were, respectively, 112.6 ± 9.7 % (n = 19, *P* > 0.05) of control (1.8 ± 0.4 Hz) and 103.5 ± 3.6 % (n = 19, *P* > 0.05) of control (7.1 ± 0.2 pA, Fig. 2d). These results indicate that LTB4 does not affect AMPA receptor-mediated sEPSC of dorsal horn neurons in either naïve or SNI rats.

**Fig. 3:**
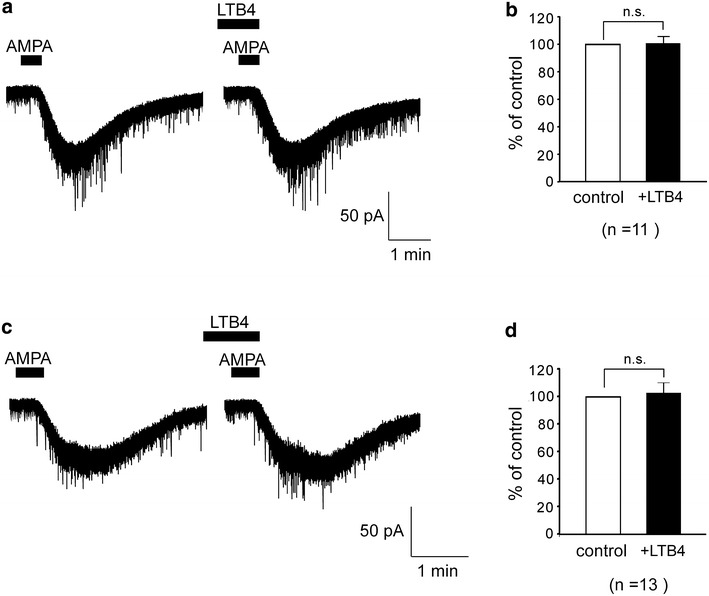
LTB4 did not affect AMPA currents in spinal dorsal horn neurons of both naïve and SNI model rats. AMPA-induced currents were not affected by application of LTB4 in either naïve (**a**) or SNI model rats (**c**) dorsal horn neurons. **b**, **d** *Bar graphs* showing the average AMPA-induced currents under the treatment with LTB4 (*black bar*), relative to those before this treatment (control, *open bar*) in naïve (**b**) and SNI model rats (**d**) (naïve; n = 11. SNI; n = 13). n.s., not significant

Next, we investigated whether LTB4 affects AMPA-induced currents in dorsal horn neurons. Exogenous application of AMPA (10 μM) for 30 s induced inward currents at a holding potential of −70 mV. After 30 s pretreatment of LTB4 (10 μM) in naïve rats, AMPA-induced inward currents did not change (n = 11, Fig. 3a). The average peak amplitude of AMPA-induced currents was 100.1 ± 4.9 % of the control (24.5 ± 3.6 pA, Fig. 3b). Next, we examined LTB4 effects in SNI model rats. AMPA-induced currents of SNI model rat also did not change (n = 13, Fig. 3c). The average peak amplitude of AMPA-induced currents was 102.9 ± 7.4 % of the control (24.5 ± 3.6 pA, Fig. 3d). The application of LTB4 did not affect AMPA-induced currents of dorsal horn neurons in either naïve or SNI rats.

### LTB4 enhanced postsynaptic NMDA currents in spinal dorsal horn neurons of SNI model rats

**Fig. 4:**
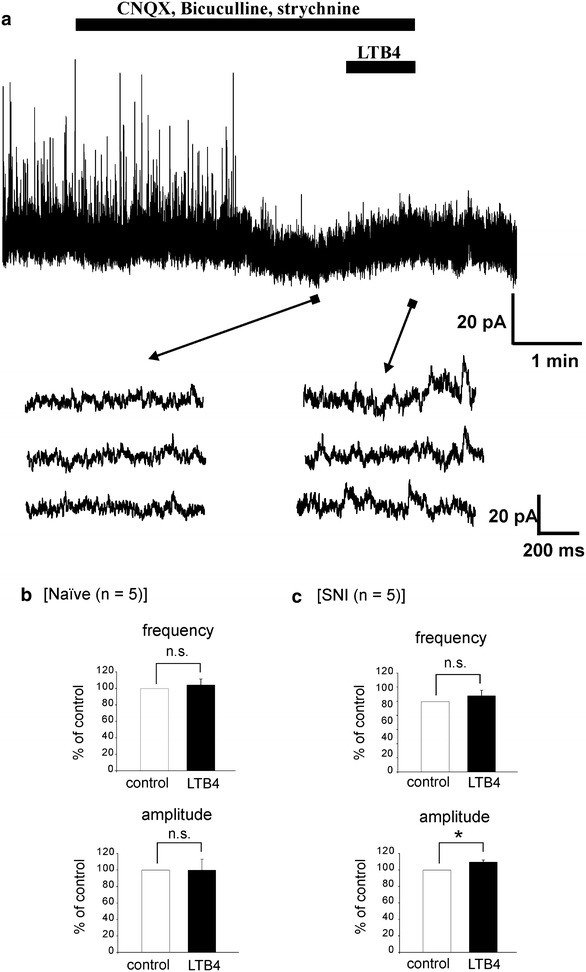
The effects of LTB4 on NMDA receptor-mediated sEPSCs. **a** NMDA receptor-mediated sEPSC has a reversed waveform with slow decay at a holding potential of +40 mV. Three consecutive traces of EPSCs are shown in an expanded *scale* in time, before (*bottom left*) and under the action of LTB4 (*bottom right*). LTB4 slightly enhanced the amplitude of NMDA receptor-mediated sEPSCs in spinal dorsal horn neurons of SNI model rats. **b**, **c** *Bar graphs* showing the average frequency and amplitude of NMDA receptor-mediated sEPSC under the treatment with LTB4 (*black bar*), relative to those before this treatment (control, *open bar*) in naïve (**b**) and SNI model rats (**c**) (naïve; n = 5. SNI; n = 5). Statistical significance between data shown by *bars* is indicated by an *asterisk*; *, *P* < 0.05; n.s., not significant

Application of LTB4 alone had no effect on AMPA receptor-mediated excitatory neurotransmission of dorsal horn neurons. However, our previous behavioral tests suggested that LTB4 contributes to neuropathic pain [25]. Next, we investigated whether LTB4 was involved in NMDA receptors response, because NMDA receptors are well known to contribute to neuropathic pain. First, we examined whether LTB4 affects NMDA receptor-mediated sEPSCs. We recorded NMDA receptor-mediated sEPSCs at a holding potential of +40 mV to completely release Mg^2+^ blockage of NMDA receptors. Under this condition, we performed the experiment during the simultaneous application of 6-cyano-7-nitroquinoxaline-2,3-dione (CNQX) (10 μM), an AMPA receptor antagonist, bicuculline (20 μM), a GABA receptor antagonist, and strychnine (2 μM), a glycine receptor antagonist, to distinguish it from IPSCs and AMPA receptor-mediated sEPSCs. In naïve rats the average increases in NMDA receptor-mediated sEPSC frequency and amplitude mediated by LTB4 (10 μM) were 104.1 ± 7.2 % and 99.8 ± 13.4 % (*n* = 5), respectively (Fig. 4b). These values were not significantly different from control (*P* < 0.05). However, we found that LTB4 slightly enhanced the amplitude of NMDA receptor-mediated sEPSCs in SNI model rats (Fig. 4a). The average increases in NMDA receptor-mediated sEPSC frequency and amplitude mediated by LTB4 were 107.8 ± 7.0 % and 109.6 ± 2.6 % (*n* = 5), respectively (Fig. 4c). This increase in amplitude was significantly large compared to control (*P* < 0.05). These results suggest LTB4 enhanced postsynaptic NMDA receptor actions in the neuropathic pain model. Therefore, we examined whether LTB4 affects postsynaptic NMDA currents in dorsal horn neurons.

**Fig. 5:**
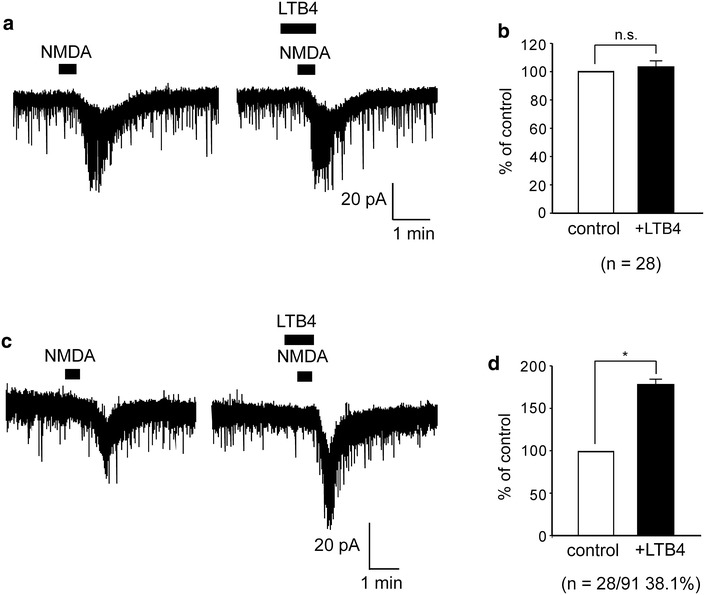
Enhanced NMDA currents by LTB4 in spinal dorsal horn neurons of SNI model rats. **a** LTB4 did not change NMDA-induced inward currents in naïve rats (n = 28). **b** *Bar graph* showing the average increase rate of NMDA-induced inward currents in naïve rats (*open bar*; control (NMDA alone), *black bar*; LTB4 + NMDA) (n = 28). **c** NMDA-induced inward currents in the presence of LTB4 were enhanced compared to without LTB4. **d** *Bar graph* showing the average increase rate of NMDA-induced inward currents in SNI model rats (*open bar*; control (NMDA alone), *black bar*; LTB4 + NMDA) (n = 28). *, *P* < 0.05; n.s., not significant

Exogenous application of NMDA (30 μM) for 30 s induced inward currents at a holding potential of −50 mV. After 30 s pretreatment of LTB4 (10 μM) in naïve rats, NMDA-induced inward currents did not change (n = 28, Fig. 5a). The average peak amplitude of NMDA-induced currents was 103.6 ± 3.9 % of the control (8.8 ± 1 pA, Fig. 5b). In the dorsal horn neurons of SNI model rats, we found that NMDA-induced currents increased in the presence of LTB4 in 28 neurons among 91 neurons with NMDA-induced currents (38.1 %, Fig. 5c). The average-peak amplitude of NMDA-induced currents was 179.3 ± 5.8 % of control (8.8 ± 0.8 pA, Fig. 5d). These findings suggest that LTB4 enhanced NMDA currents in spinal dorsal horn neurons in SNI model rats, but not in the naïve rats.

### A selective BLT1 antagonist inhibits NMDA currents induced by LTB4

**Fig. 6:**
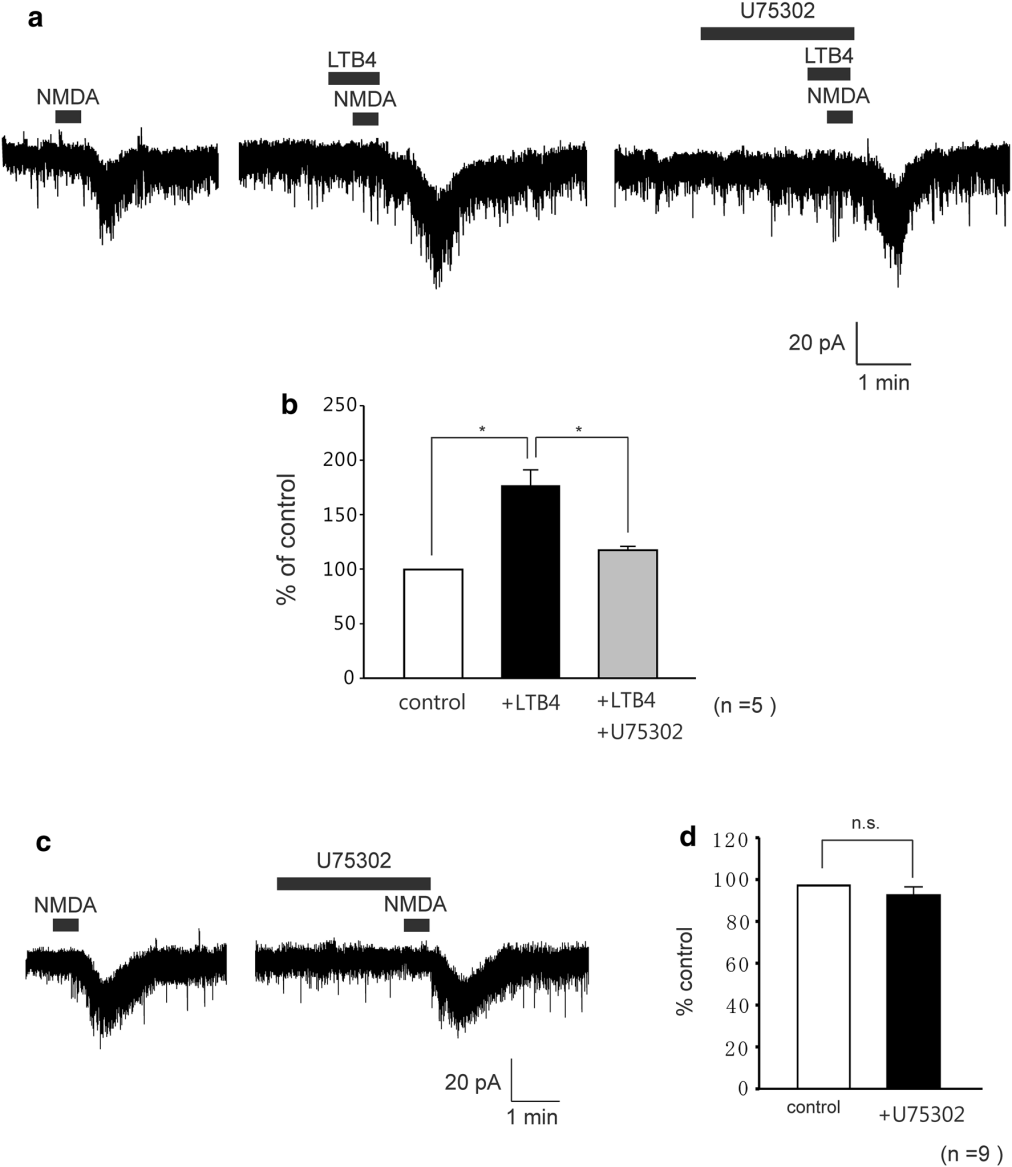
A selective BLT1 antagonist inhibited LTB4-induced enhancement of NMDA currents. **a** Enhancement of NMDA-currents by LTB4 was significantly inhibited in the presence of the selective BLT1 antagonist, U75302 (20 μM). **b** *Bar graph* showing the average increase rate of NMDA-induced inward currents by NMDA alone as control (*open bar*) and under the treatment with LTB4 (*black bar*) and in the presence LTB4 during application U75302 (*gray bar*), (n = 5). **c** U75302 had no effect on NMDA-induced currents (n = 9). **d** *Bar graph* showing the average increase rate of NMDA-induced inward currents by NMDA alone as control (*open bar*) and under the treatment with U75302 (*black bar*).  *, *P* < 0.05

BLT1 is a high affinity receptor for LTB4. To assess if the effects of LTB4 on NMDA-induced currents in dorsal horn neurons are mediated by BLT1, we investigated the effects of the presence of a selective BLT1 antagonist (U75302). In these experiments, U75302 (20 μM) was applied 2 min before treatment with LTB4 (10 μM), and the drug remained present while the agonist was being applied and during subsequent stimulation with NMDA (30 μM). The LTB4-induced enhancement of NMDA currents was significantly inhibited by U75302 (n = 5, Fig. 6a). The average peak amplitude of NMDA-induced currents in the absence and the presence of U75302 respectively were 177 ± 14.4 % and 117.6 ± 3.5 % of control (10.7 ± 2.8 pA, Fig. 6b). In order to exclude the possible non-specific effect of U75302, we found that U75302 itself had no significant effect on NMDA-induced currents (n = 9, Fig. 6c). The average-peak amplitude of NMDA-induced currents was 92.5 ± 4.5 % of control (14.9 ± 1.2 pA, Fig. 6d). These findings suggest that LTB4 exerts its actions through BLT1, and enhances NMDA-induced currents.

### Involvement of G-proteins in the LTB4 enhancement of NMDA currents

**Fig. 7:**
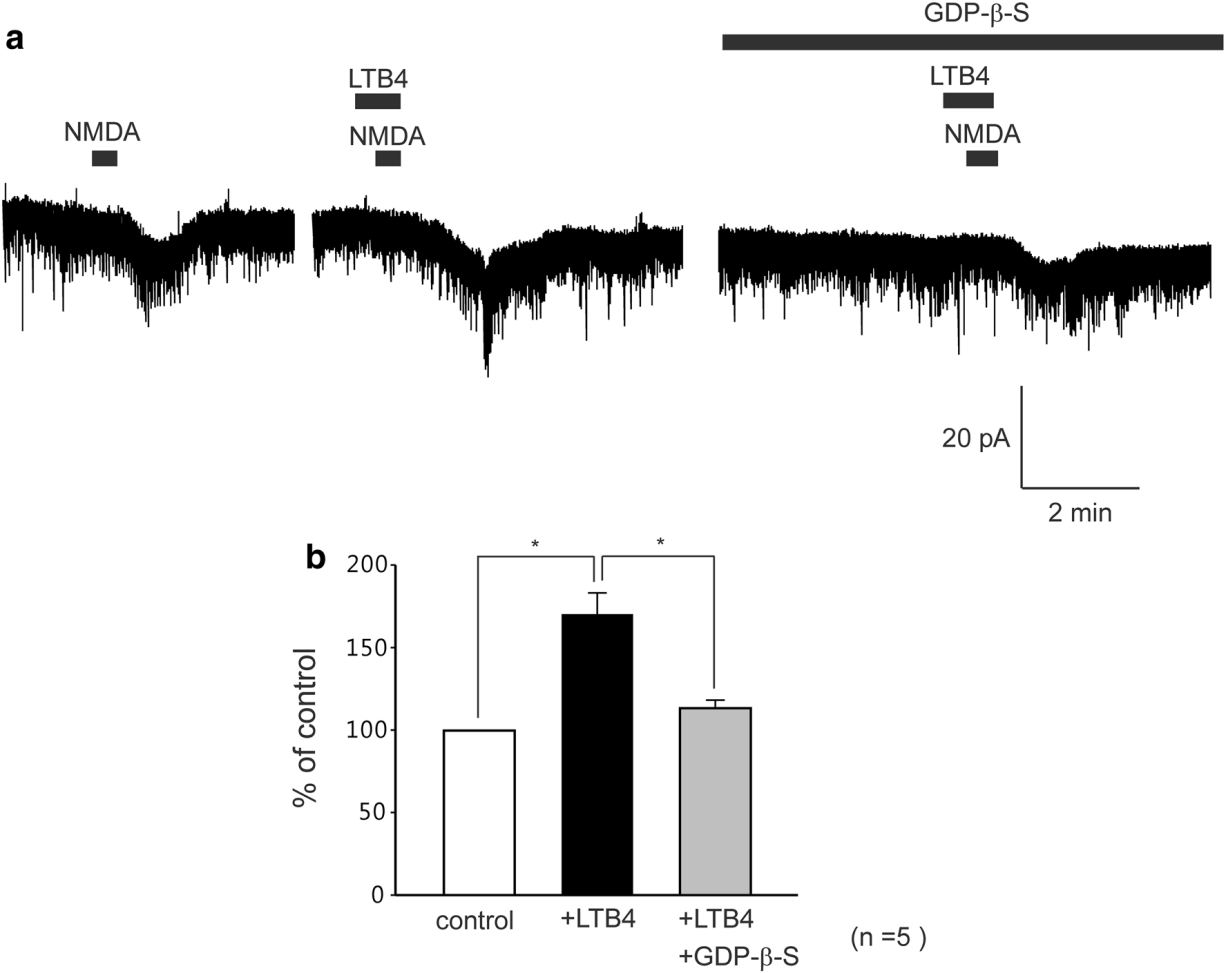
Involvement of G-proteins in the LTB4-induced enhancement of NMDA currents. NMDA-induced currents were recorded with a potassium gluconate pipette solution containing GDP-β-S (2 mM). **a** NMDA-induced currents were recorded with potassium gluconate pipette solution containing GDP-β-S. **b** *Bar graphs* showing the average increase rate of NMDA-induced inward currents by alone NMDA as control (*open bar*) and under the treatment with LTB4 (*black bar*) and in the presence LTB4 after 30 min (*gray bar*), (n = 5). *, *P* < 0.05

To examine the involvement of G-proteins in the increased NMDA-induced current by LTB4, GDP-β-S (2 mM), a non-hydrolysable analogue of GDP that competitively inhibits G-proteins, was added to the pipette solution. When LTB4 (10 μM) and NMDA (30 μM) were applied just after establishing the whole-cell configuration with pipettes containing potassium gluconate and GDP-β-S, NMDA-induced currents were clearly observed. However, when LTB4 and NMDA were again applied 30 min later, it significantly suppressed NMDA-induced currents (n = 5, Fig. 7a). The average peak amplitudes of NMDA-induced currents respectively were 170.2 ± 13.1 % and 113.5 ± 4.8 % of control (8.2 ± 1.1 pA, Fig. 7b). These findings suggest that the increased NMDA-induced currents by LTB4 were mediated through the activation of G-proteins.

## Discussion

In the present study, we examined the effects of LTB4 in the spinal dorsal horn neurons in laminar III–IV using whole-cell patch-clamp recording. The main findings are: (1) The frequency and amplitude of AMPA receptor-mediated sEPSC and AMPA-induced currents in dorsal horn neurons were not affected by application of LTB4 in either naïve or SNI model rats. (2) In contrast, the perfusion of LTB4 enhanced both the amplitude of NMDA receptor-mediated sEPSC and exogenous NMDA-induced inward currents in spinal dorsal horn neurons of SNI model rats. However, this enhancement of LTB4 on NMDA responses was not observed in naïve rats. (3) The increase of NMDA-induced inward currents was inhibited by a LTB4 antagonist, U75302, as well as a GDP-β-S, a G-protein inhibitor.

We performed blind whole-cell patch-clamp recordings from laminae III-IV dorsal horn neurons. Expression of the LTs receptor had not been examined in the spinal cord until we recently showed that the subfamily of LTs receptors, BLT1 and CysLT1, are expressed in the gray matter in the spinal cord [25]. BLT1 was localized only in neurons, while CysLT1 was localized in only microglia. One of the important findings was that the expression of BLT1 increased in the spinal cord of SNI model rats at 7 days after the peripheral nerve injury. The signal intensity of BLT1 mRNA in the dorsal horn was very low in naïve rats and it was significantly elevated in SNI model rats (Fig. 1). Because a majority of neurons expressing increased BLT1 mRNA were located in laminae III–IV, we endeavored to record the neuronal activity in laminae III–IV after peripheral nerve injury.

LTs are lipid mediators with a proinflammatory profile that have been implicated in the pathogenesis of several types of inflammation [28]. For example, the blood and synovial fluids of patients with rheumatoid arthritis contain higher levels of LTB4 than found in healthy subjects [29]. LTB4 is known as a potent neutrophil chemotactic agent. It is believed that in rheumatoid arthritis, neutrophils infiltrate synovial fluids and produce LTB4 inducing the inflammatory condition. Several studies have demonstrated that LTs are involved in peripheral inflammatory pain [30–32]. Bisgaard et al. demonstrated a reduction of the pain threshold in humans after intracutaneous deposition of LTB4 [30]. Also, both a LTB4 antagonist and BLT1 knockout mice showed reduced pain behaviors in inflammatory pain models [33]. It is well known that nerve growth factor (NGF) is up-regulated in inflammatory tissue and sensitizes nociceptors [34], leading to thermal hyperalgesia [35]. It has also been reported that NGF increased LTB4 in the rat paw skin. These results suggest the participation of LTB4 in NGF-induced local thermal hyperalgesia [36]. Other study groups have reported that intrathecal administration of LTB4 leads to thermal hyperalgesia [37]. These previous reports indicate that LTs have important roles in chronic pain at peripheral tissues, especially inflammatory pain. However, few reports have suggested that LTs are involved in the neuropathic pain via central sensitization in the spinal cord.

Our previous study showed that 5-LO and FLAP mRNAs increased in spinal microglia after SNI surgery [25]. 5-LO is the most important enzyme in the synthesis of LTs. FLAP enhances the ability of 5-LO to interact with its substrate. This finding suggests that LTs are synthesized in the activated spinal microglia after SNI. Together with the increase of BLT1 in spinal dorsal horn neurons after SNI, these findings suggest a possible interaction of activated microglia and neurons in the spinal cord. The purpose of this study was to demonstrate whether LTB4 affects the neuronal activity in dorsal horn neurons by using whole cell patch-clamp recordings. We found that bath application of LTB4 did not increase AMPA receptor-mediated sEPSCs or membrane potential. In contrast, LTB4 significantly enhanced both the amplitude of NMDA receptor-mediated sEPSCs and exogenous NMDA-induced inward currents in SNI model rats. LTB4 application did not affect sEPSC and NMDA-induced currents in naïve rats. It is probably because the expression of the BLT1 in naïve rats was very low as shown in Fig. 1.

Ionotropic glutamate receptors in the spinal dorsal horn have emerged as targets of analgesics during the last decade. It is widely known that activation of NMDA receptors is very important and essential step in both initiating and maintaining activity-dependent central sensitization and critically contributes to the development of pain hypersensitivity after peripheral tissue damage or nerve injury [38, 39]. In this study, the enhancements of NMDA-induced currents and sEPSC by LTB4 were demonstrated using the patch-clamp technique, which was consistent with the previous morphological data [25]. Moreover, the increase of NMDA current was significantly reduced by the BLT1 selective antagonist. Together, this data suggests that LTB4 increases after peripheral nerve injury, binds BLT1 in dorsal horn neurons and leads to the enhancement of excitatory neurotransmission via modulation of NMDA receptor.

The findings in this study suggest that LTB4 did not affect AMPA receptor at either the pre- or the post-synaptic sites in the dorsal horn. The reason why LTB4 has excitatory effects on NMDA-mediated neuronal activity and not on AMPA-mediated activity is unknown. To clarify this point, the precise downstream signaling after BLT1 activation is necessary [40, 41]. Increased NMDA-induced currents by LTB4 were completely blocked by the G-protein inhibitor GDP-β-S in the pipette solution, suggesting that BLT1 activation by LTB4 could enhance the sensitivity of NMDA receptors through intracellular G-proteins. This is a fundamental finding and requires more research on the precise mechanisms of signaling pathways to NMDA receptor subunits.

We previously reported that the continuous intrathecal injection of 5-LO inhibitors and LTs receptor antagonists significantly suppressed the development of mechanical hypersensitivity after SNI, but late application from 6 days after injury for 1 week did not reverse the mechanical hypersensitivity [25]. This finding indicates that LTB4 has a mechanistic role in neuropathic pain development. In this study, we performed the patch-clamp experiment on the spinal cord of rats 7–10 days after SNI. This timing was set to match the peak time point of increase of the expression of the BLT1 receptor in dorsal horn neurons. We should note the ineffectiveness in behavioral experiments in vivo, despite the fact that the BLT1 antagonist significantly suppressed the NMDA-induced current in dorsal horn neurons during this time period. It must be considered that the concentration of the antagonist in vivo is uncertain when it is intrathecally delivered. It is not easy to compare the conditions between intrathecal application into living animals and bath application onto a slice preparation. Another point which should be considered is that multiple molecules or pathways may be activated and contribute to the pain hypersensitivity in this period. For example, spinal astrocytes may be an important player that release proinflammatory cytokines and chemokines to enhance and prolong neuropathic pain [42, 43]. These molecules from astrocytes might increase the excitability of dorsal horn neurons in addition to lipid mediators including LTB4. Total net effects on dorsal horn neurons should reflect the changes in pain behaviors.

Under physiological conditions, sensory modalities are associated with dorsal horn lamination. Consistent with this pattern of afferent termination, dorsal horn second order neurons in the superficial laminae mainly receive nociceptive input while neurons in deeper dorsal horn laminae mostly convey non-nociceptive (lamina III) or converged input (laminae IV–VI) [44, 45]. However, in pathological conditions such as peripheral nerve injury, previous studies have indicated that laminae III–IV neurons with dorsal column nuclei showed dynamic changes and may have a role in the abnormal processing of input in the spinal cord which leads to mechanical hypersensitivity [46–48]. The enhancement of NMDA-mediated responses via BLT1 in SNI rats may be involved in the sensitization in laminae III–IV and result in the increase of neuronal activity, leading to neuropathic pain behaviors.

Currently, little is known about the mechanisms that induce LT synthetases in spinal microglia, or what leads the BLTl to be expressed in dorsal horn neurons. LTB4 in the spinal cord must be an important mediator in neuropathic pain, like other lipid mediators, prostaglandins and lysophosphatidic acid [3, 21]. It is also possible that other types of cells in the spinal cord produce LTB4 or other LTs after peripheral nerve injury, because previous papers using cell cultures reported the synthesis of LTB4 in astrocytes, oligodendrocytes and endothelical cells [49–51]. We believe that lipid mediators such as LTB4 might be important players in neuron-glia interaction and have a role in pain hypersensitivity via activation of NMDA-mediated responses in the dorsal horn neuron after peripheral nerve injury. The investigation of LTB4 and BLT1 may not only lead to a deeper understanding of intractable neuropathic pain, but also have the potential to find therapeutic agents in the future.

## Conclusion

Our findings showed that LTB4, which may originate from microglia, could activate BLT1 receptors, which are expressed on the membrane of spinal dorsal horn neurons during neuropathic pain. LTB4 enhanced both the amplitude of NMDA receptor-mediated sEPSC and exogenous NMDA-induced inward currents in dorsal horn neurons of SNI model rats. The enhancement of NMDA currents is through intracellular G-proteins; however the detailed mechanisms of the downstream signaling and the glia-neuron interaction in the spinal cord need further study in order to be clarified. We believe that the LTB4-BLT1 mechanism in the spinal cord may be involved in central sensitization after peripheral nerve injury.

## Methods

All of the experimental procedures involving the use of animals were approved by the Ethics Committee on Animal Experiments, Kansai University of Health Sciences and the Hyogo College of Medicine Committee on Animal Research, and were in accordance with the United Kingdom Animals (Scientific Procedures) Act of 1986 and associated guidelines. Every effort was made to minimize animal suffering and reduce the number of animals used.

### Animal procedures

Male adult Sprague–Dawley rats (4–5 weeks of age, 130–200 g) were divided into neuropathic pain model and naïve rat groups. Neuropathic pain model rats were anesthetized with sodium pentobarbital (50 mg kg^−1^, ip) and received SNI in their hindlimbs [52]. Briefly, the sciatic nerve was exposed at the level of its trifurcation into the sural, tibial, and common peroneal nerves. Each of the tibial and common peroneal nerves were tightly ligated by silk and then completely severed in between, leaving the sural nerve intact. SNI model rats were used in experiments 7–10 days after surgery.

### Reverse transcription-polymerase chain reaction (RT-PCR) and in situ hybridization histochemistry

Methods of RT-PCR and in situ hybridization histochemistry were described in detail in our previous paper [25].

### Spinal cord slice preparations

The methods used for obtaining adult rat spinal cord slice preparations have been described previously [53]. In brief, adult rats were deeply anesthetized with urethane (1.2 g/kg, IP), and then lumbosacral laminectomy was performed. The lumbosacral spinal cord (L1–S3) was removed and placed in pre-oxygenated sucrose-artificial cerebrospinal fluid (ACSF) at 1–3 °C. Sucrose-ACSF contained the following (in mM): 223 sucrose, 25 NaHCO_3_, 1.2 NaH_2_PO_4_, 3.6 KCL, 2 CaCl_2_, 1 MgCl_2_, 0.4 ascorbic acid, 2 pyruvate, 11 glucose and pH 7.4 [54]. Immediately after the removal of the spinal cord, the rats were given an overdose of urethane and were then killed by exsanguination. The pia-arachnoid membrane was removed after cutting all of the ventral and dorsal roots near the root entry zone. The spinal cord was mounted on a microslicer and then a 600-μm-thick transverse slice was cut from the lumbar segment which mainly L4 or L5 root entries. The slice was placed on a nylon mesh in the recording chamber, which had a volume of 0.5 ml, and then perfused at a rate of 10–15 ml/min with Krebs solution saturated with 95 % O_2_ and 5 % CO_2_, and maintained at 36 ± 1 °C. The Krebs solution contained the following (in mM): 117 NaCl, 3.6 KCl, 2.5 CaCl_2_, 1.2 MgCl_2_, 1.2 NaH_2_PO_4_, 25 NaHCO_3_ and 11 glucose, pH 7.4.

### Patch-clamp recordings from spinal dorsal horn neurons

Blind whole-cell patch-clamp recordings were made from spinal dorsal horn neurons (lamina III–IV) with patch-pipette electrodes having a resistance of 5–10 MΩ. The patch-pipette solution used to record EPSCs was composed of the following (in mM): 135 potassium gluconate, 5 KCl, 0.5 CaCl_2_, 2 MgCl_2_, 5 EGTA, 5 HEPES and 5 ATP-Mg, pH 7.2. Recording of NMDA receptor-mediated sEPSCs was performed using an electrode solution composed of the following (mM): Cs_2_SO_4_ 110, tetraethylammonium 5, CaCl_2_ 0.5, MgCl_2_ 2, EGTA 5, ATP-Mg 5 and HEPES–KOH 5; pH 7.2 (305 mOsm). Membrane potentials were held at −70 mV in voltage-clamp mode. After making a gigaohm seal, the membrane patch was ruptured by a brief period of more negative pressure, thus resulting in a whole cell configuration. Signals were acquired with a patch-clamp amplifier (Axopatch 200B; Molecular Devices, Sunnyvale, CA, USA). Data were digitized with an analog-to-digital converter (Digidata 1440A; Molecular Devices) and stored on a personal computer using the pCLAMP 10 data acquisition program (Molecular Devices). In this study, the exogenous NMDA currents were recorded at −50 mV, and the exogenous AMPA currents were recorded at −70 mV. The frequency and amplitude of the sEPSCs when exposed to LBT4 were analyzed at −70 mV (AMPA receptor) and +40 mV (NMDA receptor). Data were analyzed using Mini Analysis 6.0 software (Synaptosoft, Fort Lee, NJ, USA) and the pCLAMP 10 data acquisition program. EPSCs were detected with Mini Analysis by setting the following parameters: amplitude threshold, 5 pA and area threshold, 20 pA × ms with their fast rise time and decay curve. Spinal dorsal horn neurons were viable for up to 24 h in slices perfused with pre-oxygenated Krebs solution, although all of the recordings described here were obtained within 12 h. Whole-cell patch-clamp recordings were stable for up to 4 h. The membrane potentials were not corrected for the liquid junction potential between the Krebs and patch-pipette solutions.

### Application of the drugs

Drugs were dissolved in Krebs solution and applied by perfusion via a three-way stopcock without any change in the perfusion rate or the temperature. The time necessary for the solution to flow from the stopcock to the surface of the spinal cord was ~30 s. The drugs used in this study were LTB4 (5S,12R-dihydroxy-6Z,8E,10E,14Z-eicosatetraenoic acid), BLT1 receptor antagonist U75302 (6-(6-(3R-hydroxy-1E,5Z-undecadien-1-yl)-2-pyridinyl)-1,5S-hexanediol) (Sigma, Poole, UK), GDP-β-S (Sigma, St Louis, MO, USA), AMPA, NMDA, CNQX, bicuculline, and strychnine (Sigma-Aldrich, St. Louis, MO, USA). AMPA and NMDA were first dissolved in distilled water at 1000 times the concentration to be used, while CNQX, bicuculline, and strychnine were first dissolved in dimethyl sulfoxide at 1000× the concentration to be used. They were then stored at −20 °C and were diluted to the desired concentration in Krebs solution immediately before use. LTB4 and U75302 were stored in ethanol at −20 °C. The ethanol was evaporated from the drugs under a gentle stream of nitrogen immediately before use.

### Statistical analysis

All numerical data were expressed as the mean ± S.E.M. Paired Student’s t tests were used to determine the statistical significance between means. *P* < 0.05 was considered significant for these tests. For the electrophysiological data, “n” refers to the number of neurons studied.
